# Sickle Cell Trait and Kidney Disease in People of African Ancestry With HIV

**DOI:** 10.1016/j.ekir.2021.12.007

**Published:** 2021-12-13

**Authors:** Rachel K.Y. Hung, Elizabeth Binns-Roemer, John W. Booth, Rachel Hilton, Julie Fox, Fiona Burns, Mark Harber, Andrew Ustianowski, Lisa Hamzah, James E. Burns, Amanda Clarke, David A. Price, Stephen Kegg, Denis Onyango, Beatriz Santana-Suarez, Lucy Campbell, Kate Bramham, Claire C. Sharpe, Caroline A. Sabin, Cheryl A. Winkler, Frank A. Post, GEN-AFRICA Study Group, John Booth, Anele Waters, James Hand, Chris Clarke, Sarah Murphy, Maurice Murphy, Marion Campbell, Amanda Clarke, Celia Richardson, Alyson Knott, Gemma Weir, Rebecca Cleig, Helena Soviarova, Lisa Barbour, Tanya Adams, Vicky Kennard, Vittorio Trevitt, Rachael Jones, Jeremy Levy, Alexandra Schoolmeester, Serah Duro, Rachel Hilton, Julie Fox, May Rabuya, Lisa Hamzah, Deborah Jordan, Teresa Solano, Hiromi Uzu, Karen Williams, Julianne Lwanga, Linda Ekaette Reid-Amoruso, Hannah Gamlen, Robert J. Stocker, Fiona Ryan, Anele Waters, Karina Mahiouz, Tess Cheetham, Claire Williams, Achyuta Nori, Caroline Thomas, Sivaraj Venkateshwaran, Jessica Doctor, Andrea Berlanga, Frank Post, Beatriz Santana-Suarez, Leigh McQueen, Priya Bhagwandin, Lucy Campbell, Bee Barbini, Emily Wandolo, Tim Appleby, Deborah Jordan, Lois Driver, Sophy Parr, Hongbo Deng, Julie Barber, Andrew Crowe, Chris Taylor, Mary Poulton, Vida Boateng, Marie-Pierre Klein, Caitlin O’Brien, Samuel Ohene-Adomako, Christian Buckingham, Daniel Trotman, Killian Quinn, Kate Flanagan, Verity Sullivan, Holly Middleditch, Itty Samuel, Elizabeth Hamlyn, Candice McDonald, Ana Canoso, Emeka Agbasi, Maria Liskova, Sarah Barber, Amanda Samarawickrama, Zoe Ottaway, Claire Norcross, Amelia Oliveira, Kate Bramham, Jane Minton, Gary Lamont, Ruby Cross, Gaushiya Saiyad, Shadia Ahmed, Rebecca Ashworth, Nicola Window, J. Murira, Khine Phyu, Andrew Ustianowski, Gabriella Lindergard, Jonathan Shaw, Sarah Holland, Claire Fox, Jan Flaherty, Margaret-Anne Bevan, Valerie George, David Chadwick, Marie Branch, Pauline Lambert, Adele Craggs, Sarah Pett, Hinal Lukha, Nina Vora, Marzia Fiorino, Maria Muller Nunez, Deirdre Sally, James E. Burns, Erica Pool, Rebecca Matthews, David Ashley Price, Tara Stothard, Bijal Patel, Ian McVittie, Ciara Kennedy, Uli Shwab, Brendan Payne, Sarah Duncan, Jill Dixon, Mathias Schmid, Adam Evans, Christopher Duncan, Ewan Hunter, Yusri Taha, Natasha Astill, Cheryl Winkler, Elizabeth Binns-Roemer, Victor David, Jonathan Ainsworth, Rachel Vincent, Stephen Kegg, Chloe Saad, Sarah Skinner, Hocine Azzoug, Judith Russell, Tarik Moussaoui, Celia Richardson, Emily Mabonga, Donna Ward, J. Francoise, W. Larbi, Sue Mitchell, A. Manning, V. Russell, Fiona Burns, Mark Harber, Nnenna Ngwu, Jonathan Edwards, Nargis Hemat, Tom Fernandez, Filippo Ferro, Jorge Ferreira, Alice Nightingale, Tasha Oakes-Monger, Darwin Matila, Pedro Nogueira, Victoria Mutagwanya, Catherine Cosgrove, Lisa Hamzah, Catherine Emily Isitt, Helen Webb, Joyce Popoola, Kate Korley, Mark Mencias, Patricia Ribeiro, Rajeshwar Ramkhelawn, Sandra Oliva Lara, Sara Sajijad, Alan Winston, Jeremy Levy, Amber Shaw, Claire Petersen, Kyle Ring, Melanie Rosenvinge, Chloe Saad, Sarah Skinner, Thembi Moyo, Faith Odong, Katherine Gantert, Tina Ibe, Denis Onyango, Caroline Sabin, Teresa Hill

**Affiliations:** 1 King’s College London, London, UK; 2 Basic Research Laboratory, Frederick National Laboratory for Cancer Research and the National Cancer Institute, Frederick, Maryland, USA; 3 Barts Health NHS Trust, London, UK; 4 Guy’s and St Thomas’ NHS Foundation Trust, London, UK; 5 Royal Free London Hospital NHS Foundation Trust, London, UK; 6 Pennine Acute Hospitals NHS Foundation Trust, Manchester, UK; 7 St George’s Hospital NHS Foundation Trust, London, UK; 8 University College London, London, UK; 9 Central and North West London NHS Foundation Trust, London, UK; 10 Brighton and Sussex University Hospital NHS Trust, Brighton, UK; 11 Department of Infectious Disease, Brighton and Sussex Medical School, Brighton, UK; 12 The Newcastle Upon Tyne Hospitals, Newcastle, UK; 13 Lewisham and Greenwich NHS Trust, London, UK; 14 Africa Advocacy Foundation, London, UK; 15 King’s College Hospital NHS Foundation Trust, London, UK

**Keywords:** Africa, APOL1, HIV, kidney, SCT, sickle cell trait

## Abstract

**Introduction:**

Sickle cell trait (SCT) has been associated with chronic kidney disease (CKD) in African Americans, although evidence for its impact in Africans and people with HIV is currently lacking. We conducted a cross-sectional study investigating the association between SCT and kidney disease in people of African ancestry with HIV in the UK.

**Methods:**

The primary outcome was estimated glomerular filtration rate (eGFR) <60 ml/min per 1.73 m^2^. Secondary outcomes were eGFR <90 ml/min per 1.73 m^2^, end-stage kidney disease (ESKD; eGFR <15 ml/min per 1.73 m^2^, chronic dialysis, or having received a kidney transplant), proteinuria (protein-to-creatinine ratio >50 mg/mmol), and albuminuria (albumin-to-creatinine ratio >3 mg/mmol). Multivariable logistic regression was used to estimate the associations between SCT and kidney disease outcomes.

**Results:**

A total of 2895 participants (mean age 48.1 [SD 10.3], 57.2% female) were included, of whom 335 (11.6%) had SCT and 352 (12.2%) had eGFR <60 ml/min per 1.73 m^2^. After adjusting for demographic, HIV, and kidney risk factors including *APOL1* high-risk genotype status, individuals with SCT were more likely to have eGFR <60 ml/min per 1.73 m^2^ (odds ratio 1.62 [95% CI 1.14–2.32]), eGFR <90 ml/min per 1.73 m^2^ (1.50 [1.14–1.97]), and albuminuria (1.50 [1.09–2.05]). Stratified by *APOL1* status, significant associations between SCT and GFR <60 ml/min per 1.73 m^2^, eGFR <90 ml/min per 1.73 m^2^, proteinuria, and albuminuria were observed for those with *APOL1* low-risk genotypes.

**Conclusion:**

Our results extend previously reported associations between SCT and kidney disease to people with HIV. In people of African ancestry with HIV, these associations were largely restricted to those with *APOL1* low-risk genotypes.

## Graphical abstract

**Figure:**
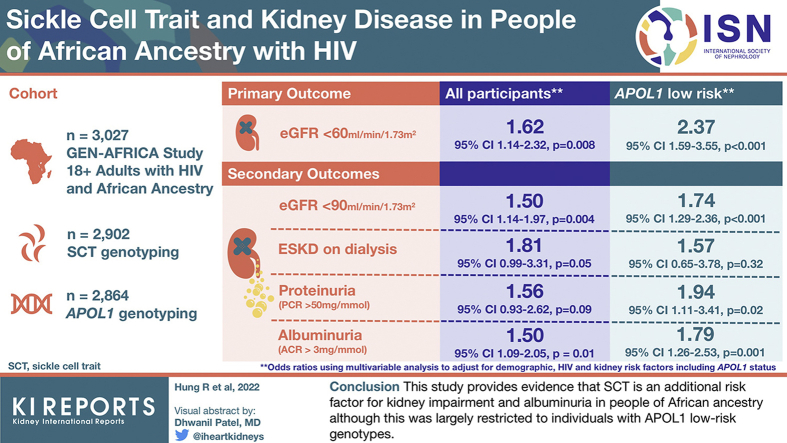

See Commentary on Page 368

CKD is an important cause of morbidity and mortality in Africa and the African diaspora.^1^^,^^2^ In black people with HIV, HIV-associated nephropathy is the most severe form of kidney disease^3^, ^4^, ^5^ and a leading cause of ESKD.^6^^,^^7^ Homozygosity (or compound heterozygosity) for *APOL1* genetic variants which provide protection against *Trypanosoma brucei* infection is a major risk factor for the development of HIV-associated nephropathy, focal and segmental glomerulosclerosis, progressive CKD, and ESKD.^8^, ^9^, ^10^ An estimated one-third of CKD and half of ESKD cases in people of African ancestry with HIV may be attributable to *APOL1* high-risk genotypes.^10^

Sickle hemoglobin (HbS), a genetic variant of the β-globin gene, provides protection against malaria.^11^^,^^12^ Homozygosity (sickle cell disease; *HbSS*) may result in CKD as a result of recurrent vaso-occlusive events in the kidney medulla; sickle cell nephropathy is characterized by proteinuria, hematuria, and urinary concentration defects, and kidney failure may follow a prolonged period of glomerular hyperfiltration.^13^ Although individuals with SCT (*HbAS*) may occasionally present with manifestations of sickle cell nephropathy, sickle cell crises are rare, and SCT carriers have normal life expectancy. In the past decade, several studies in African Americans have reported associations between SCT and CKD, albuminuria, more rapid decline in eGFR,^14^^,^^15^ and ESKD,^16^ although another study from the USA found no association between SCT and ESKD,^17^ and 2 studies from sub-Saharan Africa were unable to confirm an association between SCT and CKD.^18^^,^^19^ Moreover, the relationship between SCT and kidney disease has not been investigated in people with HIV.

We investigated the relationship between SCT and kidney impairment, ESKD, proteinuria, and albuminuria in the GEN-AFRICA (Genetic Markers of Kidney Disease Progression in People of African Ancestry with HIV in the United Kingdom) study.^20^

## Methods

The GEN-AFRICA study enrolled individuals of black ethnicity aged 18 years or over at 15 HIV clinics and 3 dialysis/kidney transplantation centers across England between May 2018 and February 2020. During a single study visit, informed consent was obtained, and demographic data including country of birth of both parents and clinical information were collected from participants using questionnaires corroborated through review of clinical records. Diabetes mellitus and hypertension were predominantly self-reported diagnoses; medical records were reviewed for those reporting but not on treatment for these conditions to verify the diagnosis. In addition, further diabetes cases were ascertained through review of medical records of those with glycosuria.

Laboratory data, including nadir and most recent CD4 cell count, viral hepatitis status, and HIV viral load were obtained from electronic patient records. Kidney function was assessed by measuring serum creatinine and urine protein-to-creatinine ratio in local laboratories, and albumin-to-creatinine ratio in stored urine samples in a central laboratory. The study was approved by a National Health Services Research Ethics Committee and Health Research Authority (18/LO/0234 and 239895).

All participants were genotyped for SCT and *APOL1* kidney risk variants (G1 and G2). Genotyping for the primary exposure variable, HbS, was performed using TaqMan SNP Genotyping Assays (Applied Biosystems/Thermo Fisher Scientific, Waltham, MA) as previously described. Analyses were restricted to those with *HbAS* and *HbAA* (noncarrier); individuals with *HbSS* were excluded. *APOL1* high-risk genotypes were defined as the presence of 2 kidney risk alleles (G1/G1, G2/G2, or G1/G2).^21^

Participants had diverse geographic backgrounds and were grouped by region of African ancestry based on self-reported country of birth of both parents: East, South, Central, and West Africa as defined by the African Union,^22^ with the exception of Angola, which was included in the Central rather than South region, or the Caribbean. Participants with parents from different African regions or outside sub-Saharan Africa or the Caribbean or whose country of birth was unknown were grouped together as “Other.” We used the 2021 CKD Epidemiology Collaboration equation (without application of the correction factor for black ethnicity to calculate eGFR).^23^ The primary outcome was eGFR <60 ml/min per 1.73 m^2^. Participants with eGFR <15 ml/min per 1.73 m^2^, a kidney transplant, or receiving chronic dialysis were categorized as having ESKD.

### Statistical Methods

The characteristics of the study population, stratified by SCT status (overall, and for those with *APOL1* high-risk and low-risk genotypes), were compared using χ^2^ tests for categorical variables and Kruskal–Wallis tests or analysis of variance for continuous variables, as appropriate. Logistic regression was used to describe the association between SCT and kidney outcomes; likelihood ratio tests were used to assess the strength of the associations at each level. It was decided *a priori* to include age and sex in all models; covariates that were associated (*P* < 0.1) with kidney disease outcomes in univariable analysis were included in the multivariable models; models for the primary outcome were additionally adjusted for HIV factors (prior AIDS, recent and nadir CD4 cell count, hepatitis B [hepatitis B antigen positive], and hepatitis C [anti–hepatitis C virus]) and comorbidities (diabetes mellitus, cardiovascular disease [a composite of any previous history of myocardial infarction, coronary artery disease, peripheral vascular disease, stroke, heart failure, and cardiomyopathy]), and *APOL1* risk allele status. We also analyzed the association between SCT and the following secondary outcomes: (i) eGFR<90 ml/min per 1.73 m^2^, (ii) ESKD, (iii) proteinuria (protein-to-creatinine ratio >50 mg/mmol, excluding those with ESKD), and (iv) albuminuria (albumin-to-creatinine ratio >3 mg/mmol, excluding those with ESKD). All other multivariable analyses were subsequently adjusted for the same covariates as the primary outcome.

As hypertension is almost invariably present in most people with CKD, the main analyses were not adjusted for hypertension. Sensitivity analyses were performed instead to assess the effects of including hypertension on the association between SCT and the primary outcome. All statistical analyses were done using STATA v16 (StataCorp, College Station, TX).

## Results

A total of 3027 individuals were enrolled in the GEN-AFRICA study. SCT genotyping was successful for 2902 (95.6%) and *APOL1* genotyping for 2864 (94.6%); 335 (11.6%) participants had SCT, and 354 (12.4%) had *APOL1* high-risk genotypes (62 had both SCT and *APOL1* high-risk genotypes); 7 participants with *HbSS* were excluded. The prevalence of SCT was 4.5%, 10.5%, 17.8%, and 21.2% among participants of South, East, West, and Central African ancestry, respectively, and 10.7% among those of Caribbean ancestry, with the highest rates observed in those from Angola, Sierra Leone, Nigeria, Cameroon, and Zambia (Supplementary Figure S1).

**Figure 1:**
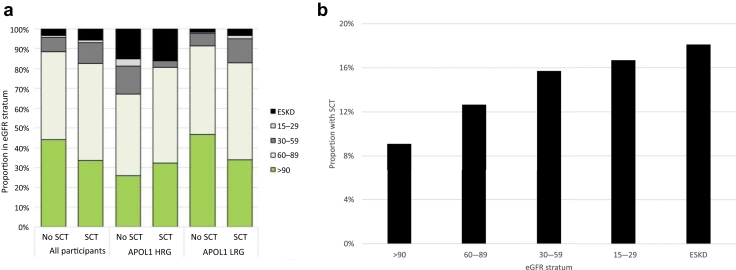
(a) Proportion of eGFR of all study participants stratified by SCT and by APOL1 status; (b) Proportion of study participants with sickle cell trait in each eGFR stratum. eGFR, estimated glomerular filtration rate; ESKD, end-stage kidney disease; SCT, sickle cell trait.

The demographic and clinical characteristics of the participants stratified by SCT status are shown in Table 1. The mean age was 48.1 (SD 10.3) years, and most had long-standing (mean 14.0 years) and well-controlled HIV (93.1% had a viral load <200 copies/ml). Participants with SCT had lower eGFR and were more likely to have proteinuria and albuminuria; they also had lower recent and nadir CD4 cell counts. Figure 1a shows the eGFR distribution for those with and without SCT. The proportion of participants with SCT increased from 9.1% to 18.1% among those with eGFR >90 ml/min per 1.73 m^2^ and ESKD, respectively (Figure 1b).

**Table 1:** Baseline characteristics of the study participants stratified by sickle cell trait status

Participant characteristics		Sickle cell trait status			
		Total (*N* = 2895)	Yes *(n* = 335)	No *(n* = 2560)	*P*-value
Age, yr	mean (SD)	48.1 (10.3)	48.3 (10.2)	48.1 (10.3)	0.70
Sex, female	*n* (%)	1655 (57.2)	182 (54.3)	1473 (57.6)	0.26
Region of ancestry					<0.001
East Africa	*n* (%)	550 (19.0)	58 (17.3)	492 (19.2)	
South Africa	*n* (%)	779 (26.9)	35 (10.4)	744 (29.1)	
Central Africa	*n* (%)	160 (5.5)	34 (10.1)	126 (4.9)	
West Africa	*n* (%)	865 (29.9)	154 (46.0)	711 (27.8)	
Caribbean	*n* (%)	355 (12.3)	38 (11.3)	317 (12.4)	
Other	*n* (%)	186 (6.4)	16 (4.8)	170 (6.6)	
HIV mode of acquisition					0.03
Heterosexual	*n* (%)	2374 (82.0)	283 (84.5)	2091 (81.7)	
MSM	*n* (%)	77 (2.7)	8 (2.4)	69 (2.7)	
Vertical	*n* (%)	232 (8.0)	15 (4.5)	217 (8.5)	
Blood products	*n* (%)	23 (0.8)	6 (1.8)	17 (0.7)	
Unknown	*n* (%)	189 (6.5)	23 (6.9)	166 (6.5)	
Time since HIV diagnosis, years	mean (SD)	14.0 (6.5)	13.6 (6.6)	14.0 (6.5)	0.28
Previous AIDS	*n* (%)	675 (24.0)	76 (23.2)	599 (24.2)	0.72
Nadir CD4 cell count, cells/mm^3^	median (IQR)	202 (80–341)	170 (47–336)	208 (84–342)	0.027
Recent CD4 cell count, cells/mm^3^	median (IQR)	560 (401–733)	511 (366–692)	566 (409–738)	0.002
On antiretroviral therapy	*n* (%)	2864 (98.9)	330 (98.5)	2534 (99.0)	0.43
HIV RNA <200 copies/ml	*n* (%)	2696 (93.1)	303 (90.4)	2393 (93.5)	0.04
HBsAg positive	*n* (%)	164 (5.7)	19 (5.8)	145 (5.7)	0.99
Anti-HCV positive	*n* (%)	39 (1.4)	2 (0.6)	37 (1.5)	0.21
Diabetes	*n* (%)	291 (10.1)	33 (10.0)	258 (10.2)	0.91
Hypertension	*n* (%)	929 (32.1)	121 (36.1)	808 (31.6)	0.10
Cardiovascular disease^a^	*n* (%)	130 (4.5)	21 (6.3)	109 (4.3)	0.10
BMI, kg/m^2^					0.93
<18.5	*n* (%)	22 (0.8)	2 (0.6)	20 (0.8)	
18.5–24.9	*n* (%)	640 (22.5)	74 (22.3)	566 (22.5)	
25–29.9	*n* (%)	1021 (35.9)	124 (37.3)	897 (35.7)	
≥30	*n* (%)	1159 (40.8)	132 (39.8)	1027 (40.9)	
Smoking status					0.43
Never	*n* (%)	2237 (77.3)	266 (79.4)	1971 (77.0)	
Ex	*n* (%)	325 (11.2)	37 (11.0)	288 (11.3)	
Current	*n* (%)	333 (11.5)	32 (9.6)	301 (11.8)	
eGFR,^b^ ml/min per 1.73 m^2^	median (IQR)	86.2 (71.6–100.8)	80.4 (68.5–96.1)	86.9 (72.1–101.3)	<0.001
≥90	*n* (%)	1245 (43.0)	113 (33.7)	1132 (44.2)	0.001
60–89	*n* (%)	1298 (44.8)	164 (49.0)	1134 (44.3)	
30–59	*n* (%)	223 (7.7)	35 (10.4)	188 (7.3)	
15–29	*n* (%)	24 (0.8)	4 (1.2)	20 (0.8)	
ESKD^c^	*n* (%)	105 (3.6)	19 (5.7)	86 (3.4)	
Urine PCR, mg/mmol^d^	median (IQR)	8.6 (6–13.4)	8.9 (6.4–14.4)	8.5 (6–13.2)	0.06
<15	*n* (%)	2220 (79.5)	244 (77.2)	1976 (79.8)	0.02
15–49	*n* (%)	428 (15.3)	47 (14.9)	381 (15.4)	
50–99	*n* (%)	76 (2.7)	19 (6.0)	57 (2.3)	
≥100	*n* (%)	66 (2.4)	6 (1.9)	60 (2.4)	
Urine ACR, mg/mmol^d^	median (IQR)	0.7 (0.4–1.9)	0.9 (0.4–2.7)	0.7 (0.4–1.7)	0.01
<3	*n* (%)	2213 (82.4)	235 (76.3)	1978 (83.2)	0.004
3–29	*n* (%)	371 (13.8)	54 (17.5)	317 (13.3)	
>30	*n* (%)	98 (3.7)	19 (6.2)	79 (3.3)	
*APOL1* risk variants					<0.001
0/1	*n* (%)	2477 (87.6)	270 (81.3)	2207 (88.4)	
2	*n* (%)	352 (12.4)	62 (18.7)	290 (11.6)	

ACR, albumin-to-creatinine ratio; BMI, body mass index; CKD-EPI, chronic kidney disease Epidemiology Collaboration; eGFR, estimated glomerular filtration rate; ESKD, end-stage kidney disease; HBsAg, hepatitis B surface antigen; HCV, hepatitis C virus; IQR, interquartile range; MSM, men who have sex with men; PCR, protein-to-creatinine ratio.

a Cardiovascular disease = composite of any previous history of myocardial infarction, coronary artery disease, peripheral vascular disease, stroke, heart failure, and cardiomyopathy.

b eGFR calculated with CKD-EPI formula (without correction for Black ethnicity).

c eGFR <15 ml/min per 1.73 m^2^ or dialysis for over 3 months or having had a kidney transplant.

d Excludes participants with ESKD.

Participants with eGFR <60 ml/min per 1.73 m^2^ were older, more likely to be male, and more likely to report an AIDS defining illness, hepatitis B and hepatitis C exposure, diabetes, and cardiovascular disease (Supplementary Table S1). They also had lower nadir and recent CD4 cell counts; 73.2% had a diagnosis of hypertension. Among those with eGFR <60 ml/min per 1.73 m^2^, both SCT (16.5%) and *APOL1* high-risk genotypes (31.7%) were significantly more frequent compared with those with preserved kidney function (SCT 10.9%, *APOL1* high-risk genotypes 9.7%). In univariable analysis, SCT, age, sex, prior AIDS, lower recent and nadir CD4 cell counts, hepatitis B, anti–hepatitis C virus, diabetes, hypertension, cardiovascular disease, and *APOL1* status were associated with eGFR <60 ml/min per 1.73 m^2^. In multivariable analysis, SCT (adjusted odds ratio 1.62 [95% CI 1.14–2.32]) and *APOL1* high-risk genotypes (4.88 [3.57–6.67]) as well as age, prior AIDS, recent CD4 cell count, diabetes, and cardiovascular disease remained associated with eGFR <60 ml/min per 1.73 m^2^ (Table 2 and Supplementary Table S2). SCT was similarly associated with eGFR <60 ml/min per 1.73 m^2^ when eGFR was calculated with correction for ethnicity (Supplementary Table S3).

**Table 2:** Associations between sickle cell trait and kidney outcomes, in all participants and by *APOL1* genotype

Outcomes	*N*	Univariable			Multivariable^a^			Interaction
		OR	95% CI	*P*-value	OR	95% CI	*P*-value	*P*-value
Primary outcome								
eGFR <60 ml/min per 1.73 m^2^								
All participants	371	1.61	1.19–2.20	0.002	1.62	1.14–2.32	0.008	0.002
- *APOL1* low risk	235	2.21	1.55–3.13	<0.001	2.37	1.59–3.55	<0.001	
- *APOL1* high risk	109	0.49	0.25–0.97	0.04	0.79	0.37–1.69	0.55	
Secondary outcomes								
eGFR <90 ml/min per 1.73 m^2^								
All participants	1723	1.56	1.23–1.98	<0.001	1.50	1.14–1.97	0.004	0.02
- *APOL1* low risk	1396	1.70	1.31–2.22	<0.001	1.74	1.29–2.36	<0.001	
- *APOL1* high risk	259	0.73	0.40–1.33	0.30	0.87	0.45–1.68	0.68	
ESKD^b^								
All participants	108	1.73	1.04–2.88	0.04	1.81	0.99–3.31	0.05	0.50
- *APOL1* low risk	47	1.97	0.94–4.11	0.07	1.57	0.65–3.78	0.32	
- *APOL1* high risk	55	1.08	0.51–2.27	0.85	2.61	1.06–6.41	0.04	
uPCR >50 mg/mmol^c^								
All participants	148	1.81	1.17–2.83	0.008	1.56	0.93–2.62	0.09	0.14
- *APOL1* low risk	119	2.12	1.32–3.42	0.002	1.94	1.11–3.41	0.02	
- *APOL1* high risk	21	0.48	0.11–2.14	0.34	0.52	0.10–2.68	0.43	
uACR >3 mg/mmol^c^								
All participants	493	1.55	1.17–2.06	0.002	1.50	1.09–2.05	0.01	0.04
- *APOL1* low risk	393	1.79	1.31–2.43	<0.001	1.79	1.26–2.53	0.001	
- *APOL1* high risk	75	0.65	0.31–1.38	0.27	0.75	0.33–1.67	0.48	

eGFR, estimated glomerular filtration rate; ESKD, end-stage kidney disease; HBsAg, hepatitis B surface antigen; HCV, hepatitis C virus; OR, odds ratio; uACR, urine albumin-to-creatinine ratio; uPCR, urine protein-to-creatinine ratio.

a Adjusted for age, sex, prior AIDS, recent CD4 cell count, nadir CD4 cell count, anti-HCV, HBsAg, diabetes, cardiovascular disease (and *APOL1* risk allele status, models for all participants only).

b eGFR <15ml/min per 1.73 m^2^ or dialysis for over 3 months or having had a kidney transplant.

c Excludes participants with ESKD.

*APOL1* high-risk genotypes interacted with SCT in the model for eGFR <60 ml/min per 1.73 m^2^ (*P*_interaction_ = 0.002); to assess the interaction, we performed a stratified analysis of eGFR <60 ml/min per 1.73 m^2^ in individuals with and without *APOL1* high-risk genotypes. Among participants with *APOL1* low-risk genotypes, those with SCT had lower eGFR and were more likely to have proteinuria and albuminuria than those without SCT, whereas among participants with *APOL1* high-risk genotypes, those with and without SCT had similar eGFR, protein-to-creatinine ratio, and albumin-to-creatinine ratio (Supplementary Table S4A and B and Figure 1a). A significant association between SCT and eGFR <60 ml/min per 1.73 m^2^ was present in the subset of participants with *APOL1* low-risk genotypes (adjusted odds ratio 2.37 [1.59–3.55]), while no association was observed among those with *APOL1* high-risk genotypes (0.79 [0.37–1.69]). Associations between SCT and other measures of kidney dysfunction (except ESKD) were also restricted to those with *APOL1* low-risk genotypes (Table 2). In our sensitivity analysis, the association between SCT and eGFR <60 ml/min per 1.73 m^2^ was minimally affected by including hypertension (Supplementary Table S5).

## Discussion

In this large African diaspora cohort of people with well-controlled HIV infection, SCT was associated with eGFR <60 ml/min per 1.73 m^2^ and albuminuria after adjustment for demographic, HIV, and kidney risk factors. *APOL1* status interacted with SCT, and analyses stratified by *APOL1* status indicated that the associations between SCT and kidney disease were largely restricted to individuals with *APOL1* low-risk genotypes. These data provide further support for the clinical significance of SCT status to kidney disease outcomes and extend observations in African Americans to people of recent African ancestry with HIV living in the United Kingdom.

The associations between SCT and eGFR <60 ml/min per 1.73 m^2^, and SCT and albuminuria, in our population are consistent with and of similar magnitude to those reported previously by Naik *et al.*^14^ in African Americans (for whom HIV status was not reported). The authors estimated that approximately 6% of kidney impairment was attributable to SCT. Longitudinal analyses have reported an increased incidence of CKD (hazard ratio 1.25) and somewhat faster eGFR decline (0.22–0.45 ml/min per 1.73 m^2^/yr) in individuals with SCT^14^^,^^15^; male sex, diabetes, hypertension, cardiovascular disease, and use of angiotensin-converting enzyme inhibitors/angiotensin receptor blockers, aspirin, and statins were associated with faster eGFR decline, while higher Hb levels were associated with slower eGFR decline in those with SCT.^15^ Data on *APOL1* status were available in one of these studies; this study reported no significant interaction by *APOL1* status for the relationship between SCT with either kidney impairment or albuminuria.^14^ The HIV status and broad geographic representation of our participants, which includes countries in which SCT and *APOL1* variants are largely absent (e.g., Ethiopia and Eritrea), both highly prevalent (e.g., Nigeria), or variably present (e.g., SCT is common and *APOL1* variants are uncommon in Cameroon and Angola), or the presence of HIV (which substantially increases the risk of kidney disease in those with *APOL1* high-risk genotypes) may have contributed to these contrasting results.

The association between SCT and ESKD is less well defined. A large study of African Americans reported an association between SCT and incident ESKD independent of age, hypertension, and diabetes (hazard ratio 2.03), a risk of similar magnitude as posed by *APOL1* high-risk genotypes.^16^ Furthermore, studies in dialysis populations have suggested that the prevalence of SCT may be about 2-fold higher than in the local black populations.^24^^,^^25^ By contrast, no evidence for an association between SCT and ESKD was found in 2 other studies,^14^^,^^17^ which is consistent with our study. However, when stratified by *APOL1* status, a significant association with ESKD was only observed for those with *APOL1* high-risk genotypes. This contrasts with a previous study in African Americans (HIV status unknown) that showed that *APOL1* status did not interact with the relationship between SCT and ESKD.^17^ This may be explained by observations that *APOL1* high-risk genotypes are more potent drivers of kidney disease (especially ESKD) in people with HIV compared with those without HIV,^8^^,^^9^ thus partially obscuring the effect of SCT. The relatively small number of ESKD cases in our cohort may also have contributed to these disparate findings.

The relationship between SCT and kidney disease has not been widely studied in sub-Saharan Africans. A cross-sectional study of 602 young Nigerian adults (137 with SCT) reported no difference in mean eGFR or proportion with kidney impairment (5.1% vs. 5.2%),^18^ while a study of 359 Congolese individuals with impaired kidney function (eGFR <90; mean age 56 years, 68 with SCT) also found no difference in the prevalence of SCT among those with kidney impairment versus those with eGFR 60-90 ml/min per 1.73 m^2^ (21% vs. 18%, *P* = 0.715).^19^ The lack of an association between SCT and kidney impairment in these African studies is not apparent but may potentially relate to differences in study populations (young people in the Nigerian study, people with kidney impairment in the Congolese study), inclusion of people without HIV, and lack of data on *APOL1* status. Moreover, continued exposure to malaria may alter the balance of adverse effects of SCT and malaria episodes on kidney function.

The strengths of this study are the large sample size, broad geographic representation of the participants, assessment of multiple kidney outcomes, healthcare setting that provides unrestricted access to antiretroviral and renal replacement therapies, and additional availability of *APOL1* genotypic status. We also acknowledge several limitations. First, we do not have data on the proportion of *HbS*, other Hb variants (e.g., *HbC*), or thalassemia status. The cross-sectional study design and use of a single creatinine reading to calculate eGFR and albuminuria measurement may not truly reflect CKD status even though most participants were clinically well and likely to have had stable kidney function. We did not consider adjustment for specific antiretrovirals to avoid channeling bias as potentially nephrotoxic drugs are generally avoided in people with or at risk of CKD. We also did not adjust for use of angiotensin-converting enzyme inhibitors or angiotensin II receptor antagonists, which may have affected proteinuria and albuminuria measurements in some participants and lack historical information on causes of CKD that are common in Africa, including infections such as schistosomiasis and malaria. Finally, the relatively young age of our participants may have rendered insufficient time for more severe kidney phenotypes to develop.

## Conclusion

In people of African ancestry with HIV, *APOL1* high-risk status is a major risk factor for CKD. This study provides evidence that SCT is an additional risk factor for kidney impairment and albuminuria in this population, although this was largely restricted to individuals with *APOL1* low-risk genotypes. Further studies are required to confirm these findings, particularly in sub-Saharan African populations.

## Disclosure

AC reports receiving personal fees and other fees from Gilead Sciences, ViiV Healthcare, and Merck Sharp & Dohme and personal fees from Theratechnologies outside of the submitted work. AU reports receiving speaker and/or advisory board fees from Gilead, Janssen, Merck Sharp & Dohme, and ViiV; however, none that overlap or relate to this work. CCS reports receiving personal fees from Novartis Pharmaceuticals, Travere Pharmaceuticals, and Napp Pharmaceuticals outside of the submitted work. RH reports receiving Speaker’s honorarium from Chiesi Ltd. JWB reports receiving personal fees from Janssen Pharmaceuticals outside of the submitted work. MH reports receiving personal fees from Trial advisory committee for Novartis, from null, outside of the submitted work. JEB reports receiving personal fees from Gilead Sciences Ltd. outside of the submitted work. FAP reports receiving grants from MRC during the conduct of the study and grants and personal fees from ViiV, Gilead, Merck Sharp & Dohme, and Janssen outside of the submitted work. All the other authors declared no competing interests.

## Appendix

### List of GEN-AFRICA Study Group

Barts Health NHS Trust, London (John Booth [principal investigator (PI)]), Anele Waters, James Hand, Chris Clarke, Sarah Murphy, Maurice Murphy); Brighton and Sussex University Hospitals, Brighton (Marion Campbell, Amanda Clarke [PI], Celia Richardson, Alyson Knott, Gemma Weir, Rebecca Cleig, Helena Soviarova, Lisa Barbour, Tanya Adams, Vicky Kennard, Vittorio Trevitt); Chelsea and Westminster Hospital, London (Rachael Jones [PI], Jeremy Levy, Alexandra Schoolmeester, Serah Duro; Guy’s and St Thomas’ Hospital, London (Rachel Hilton [PI], Julie Fox, May Rabuya, Lisa Hamzah, Deborah Jordan, Teresa Solano, Hiromi Uzu, Karen Williams, Julianne Lwanga, Linda Ekaette Reid-Amoruso, Hannah Gamlen, Robert J. Stocker, Fiona Ryan, Anele Waters, Karina Mahiouz, Tess Cheetham, Claire Williams, Achyuta Nori, Caroline Thomas, Sivaraj Venkateshwaran, Jessica Doctor, Andrea Berlanga); King’s College Hospital, London (Frank Post [chief investigator], Beatriz Santana-Suarez, Leigh McQueen, Priya Bhagwandin, Lucy Campbell, Bee Barbini, Emily Wandolo, Tim Appleby, Deborah Jordan, Lois Driver, Sophy Parr, Hongbo Deng, Julie Barber, Andrew Crowe, Chris Taylor, Mary Poulton, Vida Boateng, Marie-Pierre Klein, Caitlin O’Brien, Samuel Ohene-Adomako, Christian Buckingham, Daniel Trotman, Killian Quinn, Kate Flanagan, Verity Sullivan, Holly Middleditch, Itty Samuel, Elizabeth Hamlyn, Candice McDonald, Ana Canoso, Emeka Agbasi, Maria Liskova, Sarah Barber, Amanda Samarawickrama, Zoe Ottaway, Claire Norcross, Amelia Oliveira, Kate Bramham); Leeds Teaching Hospitals NHS Trust, Leeds (Jane Minton [PI], Gary Lamont, Ruby Cross, Gaushiya Saiyad, Shadia Ahmed, Rebecca Ashworth, Nicola Window, J Murira, Khine Phyu); North Manchester General Hospital, Manchester (Andrew Ustianowski [PI], Gabriella Lindergard, Jonathan Shaw, Sarah Holland, Claire Fox, Jan Flaherty, Margaret-Anne Bevan, Valerie George); South Tees Hospitals NHS Foundation Trust, Middlesbrough (David Chadwick [PI], Marie Branch, Pauline Lambert, Adele Craggs); Mortimer Market Centre, Central and NorthWest London NHS Foundation Trust, London (Sarah Pett [PI], Hinal Lukha, Nina Vora, Marzia Fiorino, Maria Muller Nunez, Deirdre Sally, James E. Burns, Erica Pool, Rebecca Matthews); Newcastle Upon Tyne Hospitals, Newcastle (David Ashley Price [PI], Tara Stothard, Bijal Patel, Ian McVittie, Ciara Kennedy, Uli Shwab, Brendan Payne, Sarah Duncan, Jill Dixon, Mathias Schmid, Adam Evans, Christopher Duncan, Ewan Hunter, Yusri Taha, Natasha Astill); National Cancer Institute, Frederick, USA (Cheryl Winkler, Elizabeth Binns-Roemer, Victor David); North Middlesex University Hospital, London (Jonathan Ainsworth, Rachel Vincent [PI]); Queen Elizabeth Hospital, Woolwich (Stephen Kegg [PI], Chloe Saad, Sarah Skinner, Hocine Azzoug, Judith Russell, Tarik Moussaoui, Celia Richardson, Emily Mabonga, Donna Ward, J. Francoise, W. Larbi, Sue Mitchell, A. Manning, V. Russell); Royal Free London Hospital, London (Fiona Burns [PI], Mark Harber, Nnenna Ngwu, Jonathan Edwards, Nargis Hemat, Tom Fernandez, Filippo Ferro, Jorge Ferreira, Alice Nightingale, Tasha Oakes-Monger, Darwin Matila, Pedro Nogueira, Victoria Mutagwanya); St. Georges University Hospitals, London (Catherine Cosgrove [PI], Lisa Hamzah, Catherine Emily Isitt, Helen Webb, Joyce Popoola, Kate Korley, Mark Mencias, Patricia Ribeiro, Rajeshwar Ramkhelawn, Sandra Oliva Lara, Sara Sajijad); Imperial College Healthcare NHS Trust, London (Alan Winston [PI], Jeremy Levy, Amber Shaw, Claire Petersen, Kyle Ring); University Hospital Lewisham, London (Melanie Rosenvinge [PI], Chloe Saad, Sarah Skinner, Thembi Moyo, Faith Odong, Katherine Gantert, Tina Ibe); Africa Advocacy Foundation (Denis Onyango); UK CHIC cohort (Caroline Sabin [PI], Teresa Hill).

## Supplementary Material

Supplementary File (PDF)

Supplementary File (IMAGE)

**Figure S1.** Prevalence of sickle cell trait, *APOL1* high-risk genotypes and eGFR, by country of ancestry, ppt.

**Table S1.** Baseline characteristics of the study participants stratified by the primary outcome (kidney impairment; eGFR <60 mL/min per 1.73m^2^).

**Table S2.** Full logistic regression model for the primary outcome (eGFR <60 mL/min per 1.73m^2^).

**Table S3.** Sensitivity analysis - factors associated with eGFR <60 mL/min per 1.73m^2^, with application of the eGFR correction factor for ethnicity (based on the 2009 CKD-EPI-Cr equation).

**Table S4 (A).** Baseline characteristics of the study participants with *APOL1* high-risk genotypes stratified by sickle cell trait status.

**Table S4 (B).** Baseline characteristics of the study participants with *APOL1* low-risk genotypes stratified by sickle cell trait status.

**Table S5.** Sensitivity analysis - factors associated with eGFR <60 mL/min per 1.73m^2^, with additional adjustment for hypertension.
