# Supplementary material for: Sickle Cell Trait and Kidney Disease in People of African Ancestry With HIV
Source: Kidney Int Rep. 2021 Dec 13;7(3):465–73. doi: 10.1016/j.ekir.2021.12.007 (PMC8897676; doi:10.1016/j.ekir.2021.12.007)
Supplement: Supplementary File (PDF) [file mmc1.pdf]

Supplementary material

Table S1 **Baseline characteristics of the study participants stratified by the primary outcome (eGFR <60 mL/min/1.73m^2)**

		Total	eGFR <60	eGFR ≥60	p value
		**N=3,020**	**N=371**	**N=2,649**	
Sickle cell trait	n (%)	335 (11.6)	58 (16.5)	277 (10.9)	0.002
APOL1 risk variant					<0.001
0/1 risk allele	n (%)	2,503 (87.6)	235 (68.3)	2,268 (90.3)	
2 risk alleles	n (%)	354 (12.4)	109 (31.7)	245 (9.7)	
Age, years	mean (sd)	48.1 (10.3)	54.6 (9.7)	47.2 (10.0)	<0.001
Sex, female	n (%)	1,737 (57.6)	169 (45.6)	1,568 (59.2)	<0.001
Region of ancestry					<0.001
East Africa	n (%)	585 (19.4)	51 (13.7)	534 (20.2)	
South Africa	n (%)	809 (26.8)	78 (21.0)	731 (27.6)	
Central Africa	n (%)	168 (5.6)	17 (4.6)	151 (5.7)	
West Africa	n (%)	900 (29.8)	149 (40.2)	751 (28.4)	
Caribbean	n (%)	364 (12.1)	62 (16.7)	302 (11.4)	
Other		194 (6.4)	14 (3.8)	180 (6.8)	
HIV mode of acquisition	n (%)				0.07
Heterosexual	n (%)	2,487 (82.4)	310 (83.6)	2,177 (82.2)	
MSM	n (%)	80 (2.6)	8 (2.2)	72 (2.7)	
Vertical	n (%)	236 (7.8)	18 (4.9)	218 (8.2)	
Blood products	n (%)	25 (0.8)	3 (0.8)	22 (0.8)	
Unknown	mean (sd)	192 (6.4)	32 (8.6)	160 (6.0)	
Time since HIV diagnosis, years	n (%)	14.0 (6.5)	14.4 (6.8)	14.0 (6.4)	0.20
Previous AIDS	n (%)	705 (24.1)	126 (34.6)	579 (22.6)	<0.001
Nadir CD4 cell count, cells/mm^3	median (IQR)	200.0 (80.0-340.0)	132.0 (34.0-280.0)	211.0 (89.0-347.0)	<0.001
Recent CD4 cell count, cells/mm^3	median (IQR)	558.0 (401.0-730.0)	483.5 (325.0-668.0)	567.0 (411.0-738.0)	<0.001
On antiretroviral therapy	n (%)	2,986 (98.9)	366 (98.7)	2,620 (98.9)	0.67
HIV RNA <200 copies/mL	n (%)	2,809 (93.0)	341 (91.9)	2,468 (93.2)	0.38
HBsAg positive	n (%)	169 (5.7)	28 (7.8)	141 (5.4)	0.06
Anti-HCV positive	n (%)	43 (1.5)	10 (2.8)	33 (1.3)	0.02

Diabetes	n (%)	305 (10.2)	84 (23.0)	221 (8.4)	<0.001
Hypertension	n (%)	975 (32.4)	271 (73.2)	704 (26.6)	<0.001
Cardiovascular disease	n (%)	137 (4.5)	49 (13.2)	88 (3.3)	<0.001
BMI, kg/m^2					0.84
<18.5	n (%)	23 (0.8)	4 (1.1)	19 (0.7)	
18.5-24.9	n (%)	665 (22.5)	82 (22.8)	583 (22.5)	
25-29.9	n (%)	1,059 (35.8)	124 (34.5)	935 (36.0)	
≥30	n (%)	1,208 (40.9)	149 (41.5)	1,059 (40.8)	
Smoking status					0.02
Never	n (%)	2,334 (77.3)	284 (76.5)	2,050 (77.4)	
Ex	n (%)	340 (11.3)	58 (15.6)	282 (10.6)	
Current	n (%)	346 (11.5)	29 (7.8)	317 (12.0)	
Urine PCR, mg/mmol^	median (IQR)	8.6 (6 - 13.7)	12.5 (7.2 - 37.5)	8.2 (6 - 13)	<0.001
<15	n (%)	2305 (79.2)	150 (57)	2155 (81.4)	<0.001
15-49	n (%)	460 (15.8)	61 (23.2)	399 (15.1)	
50-99	n (%)	79 (2.7)	29 (11)	50 (1.9)	
>=100	n (%)	68 (2.3)	23 (8.8)	45 (1.7)	
Urine ACR, mg/mmol^	median (IQR)	0.7 (0.4 - 1.9)	1.7 (0.6 - 13.5)	0.7 (0.4 - 1.6)	<0.001
<3	n (%)	2,296 (82.3)	146 (58.2)	2150 (84.7)	<0.001
3 - 29	n (%)	392 (14.1)	60 (23.9)	332 (13.1)	
>30	n (%)	101 (3.6)	45 (17.9)	56 (2.2)	

^ excludes participants with ESKD

For definitions and abbreviations, see footnote to Table 1

Table S2 Full logistic regression model for the primary outcome (eGFR <60 mL/min/1.73m^2)

	Univariable				Adjusted for age, sex			
	OR	95% CI	p value	LRT p value	OR	95% CI	p value	LRT p value
Sickle cell trait	1.61	1.19 - 2.20	0.002		1.65	1.20 - 2.27	0.002	
Age (years)								
<45	1			<0.001	1			<0.001
45-54.9	2.83	2.03 - 3.94	<0.001		2.78	1.97 - 3.91	<0.001	
>55	6.39	4.59 - 8.90	<0.001		6.17	4.38 - 8.70	<0.001	
Sex (female vs. male)	0.58	0.46 - 0.72	<0.001		0.66	0.52 - 0.83	<0.001	
APOL1 high risk genotype	4.29	3.30 - 5.58	<0.001					
Time since HIV diagnosis (per 5 year increment)	1.06	0.97 - 1.15	0.20					
Prior AIDS	1.82	1.44 - 2.30	<0.001					
Recent CD4 cell count, cells/mm^3								
≥500	1			<0.001				
350-499	1.26	0.95 - 1.67	0.10					
<350	2.15	1.66 - 2.79	<0.001					
Nadir CD4 cell count, cells/mm^3								
>=350	1			<0.001				
200 - 349	1.25	0.86 - 1.82	0.23					
100-199	1.66	1.14 - 2.421	0.008					
<100	2.54	1.81 - 3.55	<0.001					
HIV mode of acquisition								
Heterosexual	1			0.06				
MSM	0.78	0.37 - 1.64	0.51					
Vertical	0.58	0.35 - 0.95	0.03					
Blood products	0.96	0.28 - 3.22	0.94					
Unknown	1.41	0.94 - 2.09	0.09					

	Additionally adjusted for HIV				Additionally adjusted for renal			
	OR	95% CI	p value	LRT p value	OR	95% CI	p value	LRT p value
Sickle cell trait	1.8	1.28 - 2.52	0.001		1.62	1.14 - 2.32	0.008	
Age (years)								
<45	1			<0.001	1			<0.001
45-54.9	2.87	1.96 - 4.20	<0.001		2.76	1.85 - 4.12	<0.001	
>55	6.22	4.25 - 9.11	<0.001		5.69	3.77 - 8.59	<0.001	
Sex (female vs. male)	0.79	0.61 - 1.02	0.07		0.79	0.60 - 1.03	0.08	
APOL1 high risk genotype					4.88	3.57 - 6.67	<0.001	
Time since HIV diagnosis (per 5 year increment)								
Prior AIDS	1.52	0.15 - 2.00	0.003		1.49	1.11 - 2.01	0.008	
Recent CD4 cell count, cells/mm^3								
≥500	1			0.002	1			<0.001
350-499	1.10	0.80 - 1.52	0.55		1.18	0.84 - 1.67	0.34	
<350	1.78	1.29 - 2.45	<0.001		1.81	1.28 - 2.55	0.001	
Nadir CD4 cell count, cells/mm^3								
>=350	1			0.18	1			0.54
200 - 349	1.01	0.67 - 1.51	0.96		1.03	0.67 - 1.57	0.90	
100-199	1.09	0.72 - 1.64	0.69		1.02	0.66 - 1.58	0.93	
<100	1.41	0.96 - 2.07	0.08		1.27	0.84 - 1.91	0.25	
HIV mode of acquisition								
Heterosexual								
MSM								
Vertical								
Blood products								
Unknown								

On antiretroviral therapy	0.81	0.31 - 2.11	0.67						
HIV viral load (<200 copies/mL)	0.83	0.56 - 1.25	0.38						
HBsAg positive	1.48	0.97 - 2.26	0.07	1.24	0.77 - 1.98	0.38	1.19	0.72 - 1.97	0.50
Anti-HCV positive	2.23	1.09 - 4.56	0.03	1.81	0.77 - 4.29	0.18	1.86	0.73 - 4.72	0.19
Diabetes	3.25	2.46 - 4.31	<0.001				2.05	1.43 - 2.92	<0.001
Hypertension	7.54	5.90 - 9.64	<0.001						
Cardiovascular disease	4.42	3.06 - 6.39	<0.001				3.23	2.06 - 5.04	<0.001

LRT = likelihood ratio test

Table S3: sensitivity analysis - factors associated with eGFR <60 mL/min/1.73m^2**, with application of the eGFR correction factor for ethnicity (based on the 2009 CKD-EPI-Cr equation**

	**Univariable**				**Multivariable**			
	**OR**	**95% CI**	**p value**	**LRT p value**	**OR**	**95% CI**	**p value**	**LRT p value**
Sickle cell trait	1.67	1.16 - 2.41	0.006		1.79	1.17 - 2.75	0.008	
Age (years)				<0.001				
<45	1				1			
45-54.9	2.07	1.42 - 3.02	<0.001		1.76	1.10 - 2.81	0.02	
>55	4.18	2.87 - 6.08	<0.001		3.06	1.87 - 4.98	<0.001	
Sex (female vs. male)	0.59	0.45 - 0.77	<0.001		0.94	0.67 - 1.32	0.71	
APOL1 high risk genotype	5.67	4.21 - 7.65	<0.001		6.42	4.46 - 9.23	<0.001	
Time since HIV diagnosis (per 5 year increment)	1.1	0.99 - 1.22	0.06					
Prior AIDS	1.95	1.47 - 2.59	<0.001		1.85	1.29 - 2.66	0.001	
Recent CD4 cell count, cells/mm^3				<0.001				<0.001
≥500	1				1			
350-499	1.48	1.04 - 2.10	0.03		1.53	0.99 - 2.37	0.06	
<350	3.09	2.28 - 4.21	<0.001		2.7	1.77 - 4.12	<0.001	
Nadir CD4 cell count, cells/mm^3				<0.001				0.48
>=350	1				1			
200 - 349	1.6	0.97 - 2.64	0.07		1.1	0.62 - 1.96	0.75	
100-199	2.3	1.40 - 3.78	0.001		1.21	0.68 - 2.16	0.51	
<100	3.37	2.14 - 5.31	<0.001		1.46	0.85 - 2.52	0.17	
HIV mode of acquisition				0.03				
Heterosexual	1							
MSM	0.93	0.40 - 2.17	0.87					
Vertical	0.4	0.20 - 0.83	0.01					
Blood products	1.57	0.47 - 5.28	0.47					
Unknown	1.42	0.88 - 2.27	0.16					
On antiretroviral therapy	0.88	0.27 - 2.90	0.83					

HIV viral load (<200 copies/mL)	0.91	0.55 - 1.50	0.7			
HBsAg positive	1.29	0.76 - 2.20	0.35			
Anti-HCV positive	3.29	1.56 - 6.95	0.002	3.64	1.73 - 4.11	<0.001
Diabetes	3.78	2.75 - 5.20	<0.001	2.66	1.73 - 4.11	<0.001
Hypertension	16.69	11.39 - 24.45	<0.001			
Cardiovascular disease	5.17	3.46 - 7.72	<0.001	4.27	2.60 - 7.04	<0.001

LRT = likelihood ratio test

Table S4 (A) Baseline characteristics of the study participants with *APOL1* high risk genotypes stratified by sickle cell trait status

APOL1 high risk genotypes		Total<br>N=352	SCT<br>N=62	No SCT<br>N=290	p value
Age, years	mean (SD)	48.2 (10.0)	47.3 (8.6)	48.4 (10.3)	0.45
Sex, female	n (%)	185 (52.6)	34 (54.8)	151 (52.1)	0.69
Region of ancestry					0.39
East Africa	n (%)	11 (3.1)	1 (1.6)	10 (3.4)	
South Africa	n (%)	51 (14.5)	4 (6.5)	47 (16.2)	
Central Africa	n (%)	8 (2.3)	2 (3.2)	6 (2.1)	
West Africa	n (%)	219 (62.2)	44 (71.0)	175 (60.3)	
Caribbean	n (%)	50 (14.2)	9 (14.5)	41 (14.1)	
Other	n (%)	13 (3.7)	2 (3.2)	11 (3.8)	
HIV mode of acquisition	n (%)				0.04
Heterosexual	n (%)	285 (81.0)	52 (83.9)	233 (80.3)	
MSM	n (%)	11 (3.1)	0 (0.0)	11 (3.8)	
Vertical	n (%)	27 (7.7)	2 (3.2)	25 (8.6)	
Blood products	n (%)	3 (0.9)	2 (3.2)	1 (0.3)	
Unknown	n (%)	26 (7.4)	6 (9.7)	20 (6.9)	
Time since HIV diagnosis, years	mean (SD)	12.7 (6.4)	12.5 (6.2)	12.8 (6.4)	0.76
Previous AIDS	n (%)	86 (25.2)	10 (16.1)	76 (27.2)	0.07
Nadir CD4 cell count, cells/mm^3	median (IQR)	187 (71-316)	198 (114-320)	185 (67-316)	0.35
Recent CD4 cell count, cells/mm^3	median (IQR)	539 (386-702)	525 (407-700)	547 (378-703)	0.92
On antiretroviral therapy	n (%)	345 (98.0)	60 (96.8)	285 (98.3)	0.44
HIV RNA <200 copies/mL	n (%)	325 (92.3)	52 (83.9)	273 (94.1)	0.006
HBsAg positive	n (%)	24 (6.9)	4 (6.5)	20 (7.0)	0.88
Anti-HCV positive	n (%)	4 (1.2)	0 (0.0)	4 (1.4)	0.35
Diabetes	n (%)	34 (9.7)	6 (9.7)	28 (9.7)	0.99
Hypertension	n (%)	174 (49.4)	29 (46.8)	145 (50.0)	0.64
Cardiovascular disease	n (%)	21 (6.0)	0 (0.0)	21 (7.2)	0.03

BMI categories (kg/m^2)					0.86
<18.5	n (%)	0	0	0	
18.5-24.99	n (%)	64 (18.7)	10 (16.4)	54 (19.1)	
25-30	n (%)	138 (40.2)	26 (42.6)	112 (39.7)	
≥30	n (%)	141 (41.1)	25 (41.0)	116 (41.1)	
eGFR, mL/min/1.73m^2	median (IQR)	76.0 (54.7 - 92.1)	76.3 (64.9 - 98.6)	75.2 (52.4 - 91.3)	0.31
≥90	n (%)	95 (27.0)	20 (32.3)	75 (25.9)	0.08
60-89	n (%)	150 (42.6)	30 (48.4)	120 (41.4)	
30-59	n (%)	43 (12.2)	2 (3.2)	41 (14.1)	
15-29	n (%)	10 (2.8)	0 (0.0)	10 (3.4)	
ESKD	n (%)	54 (15.3)	10 (16.1)	44 (15.2)	
Urine PCR, mg/mmol^	median (IQR)	9 (6 - 14.8)	9.8 (7 - 14.8)	9 (6 - 14.9)	0.53
<15	n (%)	229 (76.9)	40 (76.9)	189 (76.8)	0.72
15-49	n (%)	48 (16.1)	10 (19.2)	38 (15.5)	
50-99	n (%)	9 (3)	1 (1.9)	8 (3.3)	
≥100	n (%)	12 (4)	1 (1.9)	11 (4.5)	
Urine ACR, mg/mmol^	median (IQR)	1 (0.5 - 3)	1.1 (0.4 - 2.4)	1 (0.5 - 3.3)	0.48
<3	n (%)	218 (74.7)	42 (80.8)	176 (73.3)	0.53
3 - 29	n (%)	51 (17.5)	7 (13.5)	44 (18.3)	
≥30	n (%)	23 (7.9)	3 (5.8)	20 (8.3)	

^ excludes participants with ESKD

For definitions and abbreviations, see footnote to Table 1

Table S4 (B) Baseline characteristics of the study participants with *APOL1* low risk genotypes stratified by sickle cell trait status

APOL1 low risk genotypes		Total	SCT	No SCT	p value
		**N=2,477**	**N=270**	**N=2,207**	
Age, years	mean (SD)	48.1 (10.3)	48.7 (10.4)	48.0 (10.3)	0.31
Sex, female	n (%)	1,433 (57.9)	147 (54.4)	1,286 (58.3)	0.23
Region of ancestry					<0.001
East Africa	n (%)	532 (21.5)	57 (21.1)	475 (21.5)	
South Africa	n (%)	709 (28.6)	31 (11.5)	678 (30.7)	
Central Africa	n (%)	148 (6.0)	31 (11.5)	117 (5.3)	
West Africa	n (%)	627 (25.3)	109 (40.4)	518 (23.5)	
Caribbean	n (%)	294 (11.9)	29 (10.7)	265 (12.0)	
Other	n (%)	167 (6.7)	13 (4.8)	154 (7.0)	
HIV mode of acquisition	n (%)				0.15
Heterosexual	n (%)	2,030 (82.0)	229 (84.8)	1,801 (81.6)	
MSM	n (%)	62 (2.5)	7 (2.6)	55 (2.5)	
Vertical	n (%)	204 (8.2)	13 (4.8)	191 (8.7)	
Blood products	n (%)	19 (0.8)	4 (1.5)	15 (0.7)	
Unknown	n (%)	162 (6.5)	17 (6.3)	145 (6.6)	
Time since HIV diagnosis, years	mean (SD)	14.1 (6.5)	13.9 (6.6)	14.2 (6.4)	0.48
Previous AIDS	n (%)	572 (23.8)	65 (24.7)	507 (23.7)	0.72
Nadir CD4 cell count, cells/mm^3	median (IQR)	205 (83-342)	168 (44-337)	210 (89-342)	0.009
Recent CD4 cell count, cells/mm^3	median (IQR)	564 (404-741)	510 (360-692)	570 (412-748)	0.001
On antiretroviral therapy	n (%)	2,453 (99.0)	267 (98.9)	2,186 (99.0)	0.8
HIV RNA <200 copies/mL	n (%)	2,312 (93.3)	249 (92.2)	2,063 (93.5)	0.44
HBsAg positive	n (%)	133 (5.4)	15 (5.7)	118 (5.4)	0.87
Anti-HCV positive	n (%)	31 (1.3)	2 (0.8)	29 (1.3)	0.43
Diabetes	n (%)	251 (10.2)	27 (10.2)	224 (10.2)	0.96
Hypertension	n (%)	728 (29.4)	92 (34.1)	636 (28.8)	0.08
Cardiovascular disease	n (%)	102 (4.1)	21 (7.8)	81 (3.7)	0.001

BMI categories (kg/m^2)					0.95
<18.5	n (%)	22 (0.9)	2 (0.7)	20 (0.9)	
18.5-24.99	n (%)	556 (22.8)	63 (23.5)	493 (22.7)	
25-30	n (%)	869 (35.7)	98 (36.6)	771 (35.5)	
≥30	n (%)	990 (40.6)	105 (39.2)	885 (40.8)	
eGFR, mL/min/1.73m^2	median (IQR)	87.5 (73.8 - 101.3)	80.8 (69.4 - 95.8)	88.4 (74.6 - 102.0)	<0.001
≥90	n (%)	1,125 (45.4)	92 (34.1)	1,033 (46.8)	
60-89	n (%)	1,118 (45.1)	132 (48.9)	986 (44.7)	
30-59	n (%)	173 (7.0)	33 (12.2)	140 (6.3)	
15-29	n (%)	14 (0.6)	4 (1.5)	10 (0.5)	
ESKD	n (%)	47 (1.9)	9 (3.3)	38 (1.7)	
Urine PCR, mg/mmol^	median (IQR)	8.5 (6 - 13.3)	8.7 (6.4 - 14.2)	8.4 (6 - 13.1)	0.1
<15	n (%)	1942 (79.9)	202 (77.4)	1740 (80.2)	<0.001
15-49	n (%)	371 (15.3)	37 (14.2)	334 (15.4)	
50-99	n (%)	65 (2.7)	18 (6.9)	47 (2.2)	
≥100	n (%)	52 (2.1)	4 (1.5)	48 (2.2)	
Urine ACR, mg/mmol ^	median (IQR)	0.7 (0.4 - 1.7)	0.9 (0.4 - 2.7)	0.7 (0.4 - 1.6)	0.01
<3	n (%)	1948 (83.3)	191 (75.2)	1757 (84.4)	<0.001
3 - 29	n (%)	314 (13.4)	47 (18.5)	267 (12.8)	
≥30	n (%)	73 (3.2)	16 (6.3)	57 (2.7)	

^ excludes participants with ESKD

For definitions and abbreviations, see footnote to Table 1

Table S5: sensitivity analysis - factors associated with eGFR <60 mL/min/1.73m^{2,} **with additional adjustment for hypertension**

	**Univariable**				**Multivariable**			
	**OR**	**95% CI**	**p value**	**LRT p value**	**OR**	**95% CI**	**p value**	**LRT p value**
Sickle cell trait	1.61	1.19 - 2.20	0.002		1.54	1.07 - 2.23	0.02	
Age (years)								
<45	1			<0.001	1			
45-54.9	2.83	2.03 - 3.94	<0.001		2.09	1.38 - 3.16	<0.001	
>55	6.39	4.59 - 8.90	<0.001		3.51	2.28 - 5.40	<0.001	
Sex (female vs. male)	0.58	0.46 - 0.72	<0.001		0.77	0.59 - 1.02	0.07	
APOL1 high risk genotype	4.29	3.30 - 5.58	<0.001		3.87	2.80 - 5.35	<0.001	
Time since HIV diagnosis (per 5 year increment)	1.06	0.97 - 1.15	0.20					
Prior AIDS	1.82	1.44 - 2.30	<0.001		0.43	1.05 - 1.93	0.02	
Recent CD4 cell count, cells/mm^3								0.005
$\geq$500	1			<0.001	1			
350-499	1.26	0.95 - 1.67	0.10		1.22	0.86 - 1.73	0.26	
<350	2.15	1.66 - 2.79	<0.001		1.81	1.27 - 2.59	0.001	
Nadir CD4 cell count, cells/mm^3								
>=350	1			<0.001	1			0.75
200 - 349	1.25	0.86 - 1.82	0.23		0.95	0.61 - 1.46	0.81	
100-199	1.66	1.14 - 2.421	0.008		0.95	0.61 - 1.48	0.82	
<100	2.54	1.81 - 3.55	<0.001		1.14	0.75 - 1.72	0.55	
HIV mode of acquisition								
Heterosexual	1			0.06				
MSM	0.78	0.37 - 1.64	0.51					
Vertical	0.58	0.35 - 0.95	0.03					
Blood products	0.96	0.28 - 3.22	0.94					
Unknown	1.41	0.94 - 2.09	0.09					
On antiretroviral therapy	0.81	0.31 - 2.11	0.67					
HIV viral load (<200 copies/mL)	0.83	0.56 - 1.25	0.38					
HBsAg positive	1.48	0.97 - 2.26	0.07		1.34	0.80 - 2.25	0.27	
Anti-HCV positive	2.23	1.09 - 4.56	0.03		1.76	0.66 - 4.68	0.26	
Diabetes	3.25	2.46 - 4.31	<0.001		1.53	1.06 - 2.21	0.02	
Hypertension	7.54	5.90 - 9.64	<0.001		2.29	1.45 - 3.64	<0.001	
Cardiovascular disease	4.42	3.06 - 6.39	<0.001		3.87	2.86 - 5.23	<0.001	

LRT = likelihood ratio test

Appendix: Genetic markers of chronic kidney disease in people of African ancestry with HIV (GEN-AFRICA) Study Group

Barts Health NHS Trust, London (John Booth [PI], Anele Waters, James Hand, Chris Clarke, Sarah Murphy, Maurice Murphy); **Brighton and Sussex University Hospitals, Brighton** (Marion Campbell, Amanda Clarke [PI], Celia Richardson, Alyson Knott, Gemma Weir, Rebecca Cleig, Helena Soviarova, Lisa Barbour, Tanya Adams, Vicky Kennard, Vittorio Trevitt); **Chelsea and Westminster Hospital, London** (Rachael Jones [PI], Jeremy Levy, Alexandra Schoolmeester, Serah Duro; **Guy's and St Thomas' Hospital, London** (Rachel Hilton [PI], Julie Fox, May Rabuya, Lisa Hamzah, Deborah Jordan, Teresa Solano, Hiromi Uzu, Karen Williams, Julianne Lwanga, Linda Ekaette Reid-Amoruso, Hannah Gamlen, Robert J. Stocker, Fiona Ryan, Anele Waters, Karina Mahiouz, Tess Cheetham, Claire Williams, Achyuta Nori, Caroline Thomas, Sivaraj Venkateshwaran, Jessica Doctor, Andrea Berlanga); **King's College Hospital, London** (Frank Post [CI], Beatriz Santana-Suarez, Leigh McQueen, Priya Bhagwandin, Lucy Campbell, Bee Barbini, Emily Wandolo, Tim Appleby, Deborah Jordan, Lois Driver, Sophy Parr, Hongbo Deng, Julie Barber, Andrew Crowe, Chris Taylor, Mary Poulton, Vida Boateng, Marie-Pierre Klein, Caitlin O'Brien, Samuel Ohene-Adomako, Christian Buckingham, Daniel Trotman, Killian Quinn, Kate Flanagan, Verity Sullivan, Holly Middleditch, Itty Samuel, Elizabeth Hamlyn, Candice McDonald, Ana Canoso, Emeka Agbasi, Maria Liskova, Sarah Barber, Amanda Samarawickrama, Zoe Ottaway, Claire Norcross, Amelia Oliveira, Kate Bramham); **Leeds Teaching Hospitals NHS Trust, Leeds** (Jane Minton [PI], Gary Lamont, Ruby Cross, Gaushiya Saiyad, Shadia Ahmed, Rebecca Ashworth, Nicola Window, J Murira, Khine Phyu); **North Manchester General Hospital, Manchester** (Andrew Ustianowski [PI], Gabriella Lindergard, Jonathan Shaw, Sarah Holland, Claire Fox, Jan Flaherty, Margaret-Anne Bevan, Valerie George); **South Tees Hospitals NHS Foundation Trust, Middlesbrough** (David Chadwick [PI], Marie Branch, Pauline Lambert, Adele Craggs); **Mortimer Market Centre, Central and NorthWest London NHS Foundation Trust, London** (Sarah Pett [PI], Hinal Lukha, Nina Vora, Marzia Fiorino, Maria Muller Nunez, Deirdre Sally, James E. Burns, Erica Pool, Rebecca Matthews); **Newcastle upon Tyne Hospitals, Newcastle** (David Ashley Price [PI], Tara Stothard, Bijal Patel, Ian McVittie, Ciara Kennedy, Uli Shwab, Brendan Payne, Sarah Duncan, Jill Dixon, Mathias Schmid, Adam Evans, Christopher Duncan, Ewan Hunter, Yusri Taha, Natasha Astill); **National Cancer Institute, Frederick, USA** (Cheryl Winkler, Elizabeth Binns-Roemer, Victor David); **North Middlesex University Hospital, London** (Jonathan Ainsworth, Rachel Vincent [PI]); **Queen Elizabeth Hospital, Woolwich** (Stephen Kegg [PI], Chloe Saad, Sarah Skinner, Hocine Azzoug, Judith Russell, Tarik Moussaoui, Celia Richardson, Emily Mabonga, Donna Ward, J. Francoise, W. Larbi, Sue Mitchell, A. Manning, V. Russell); **Royal Free London Hospital, London** (Fiona Burns [PI], Mark Harber, Nnenna Ngwu, Jonathan Edwards, Nargis Hemat, Tom Fernandez, Filippo Ferro, Jorge Ferreira, Alice Nightingale, Tasha Oakes-Monger, Darwin Matila, Pedro Nogueira, Victoria Mutagwanya); **St. Georges University Hospitals, London** (Catherine Cosgrove [PI], Lisa Hamzah, Catherine Emily Isitt, Helen Webb, Joyce Popoola, Kate Korley, Mark Mencias, Patricia Ribeiro, Rajeshwar Ramkhelawn, Sandra Oliva Lara, Sara Sajijad); **Imperial College Healthcare NHS Trust, London** (Alan Winston [PI], Jeremy Levy, Amber Shaw, Claire Petersen, Kyle Ring); **University Hospital Lewisham, London** (Melanie Rosenvinge [PI], Chloe Saad, Sarah Skinner, Thembi Moyo, Faith Odong, Katherine Gantert, Tina Ibe); **Africa Advocacy Foundation** (Denis Onyango); **UK CHIC cohort** (Caroline Sabin [PI], Teresa Hill)
