# Supplementary material for: Sickle Cell Trait and Kidney Disease in People of African Ancestry With HIV
Source: Kidney Int Rep. 2021 Dec 13;7(3):465–73. doi: 10.1016/j.ekir.2021.12.007 (PMC8897676; doi:10.1016/j.ekir.2021.12.007)
Supplement: Supplementary File (IMAGE) [file mmc2.pptx]

## Slide 1
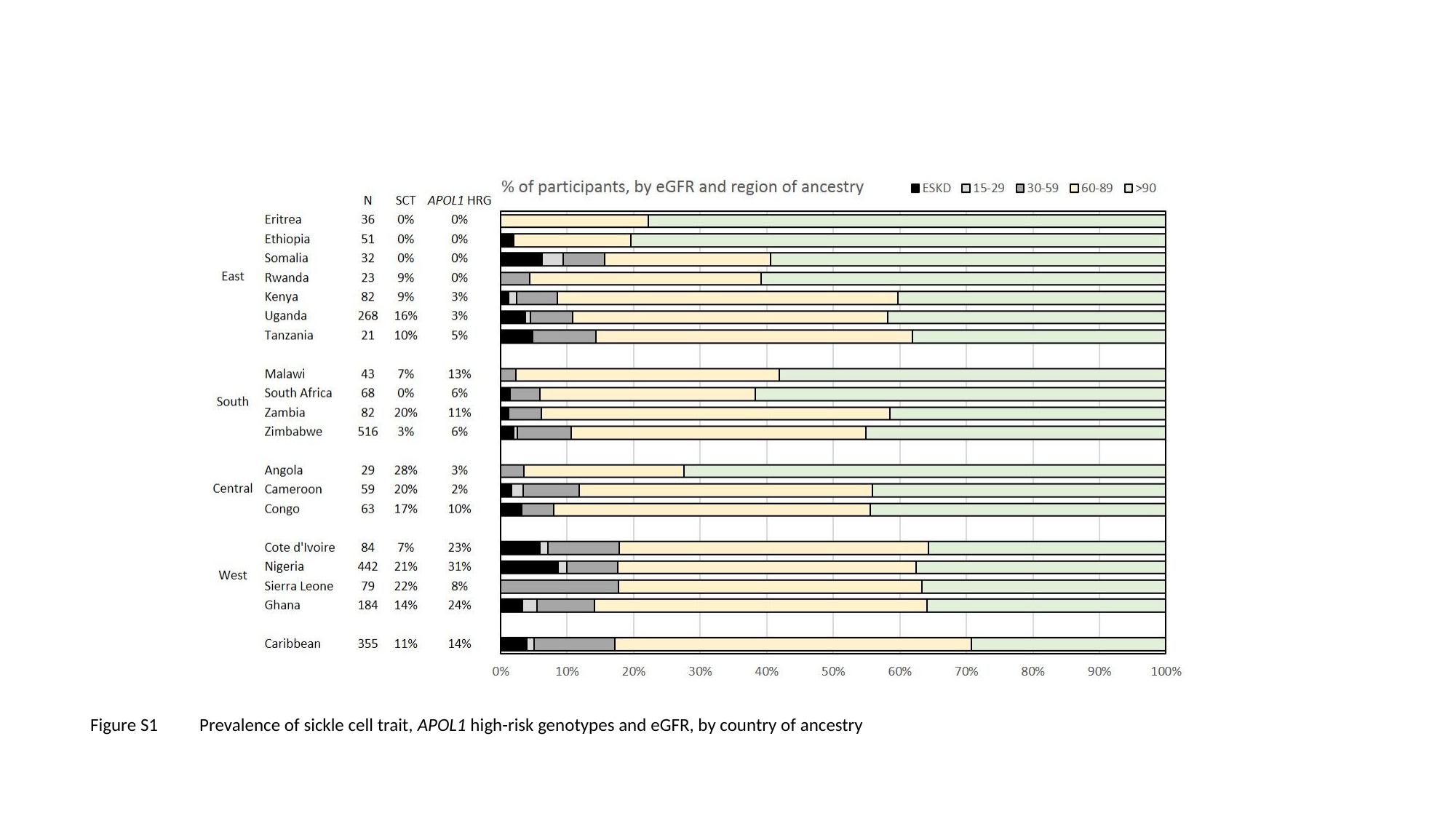

Figure S1	Prevalence of sickle cell trait, APOL1 high-risk genotypes and eGFR, by country of ancestry
